# Risk of malignancy and clinical outcomes of cyst fluid only nodules in the thyroid based on ultrasound and aspiration cytology

**DOI:** 10.1002/dc.24323

**Published:** 2019-10-18

**Authors:** Risa Kanematsu, Mitsuyoshi Hirokawa, Miyoko Higuchi, Ayana Suzuki, Hitomi Aga, Aki Tanaka, Naoki Yamao, Toshitetsu Hayashi, Seiji Kuma, Akira Miyauchi

**Affiliations:** ^1^ Department of Clinical Laboratory Kuma Hospital Kobe Hyogo Japan; ^2^ Department of Diagnostic Pathology and Cytology Kuma Hospital Kobe Hyogo Japan; ^3^ Department of Surgery Kuma Hospital Kobe Hyogo Japan

**Keywords:** Bethesda system, cyst fluid only, foamy cells, papillary thyroid carcinoma, Thyroid aspiration cytology

## Abstract

**Background:**

The number of extensive studies focusing on cyst fluid only (CFO) thyroid nodules is limited, and the risk of malignancy (ROM) in CFO nodules has not been well‐established. Thus, the purpose of this study was to investigate CFO nodules using cytology and ultrasound. In addition, we sought to define the ROM and determine the recommended clinical management of CFO nodules.

**Methods:**

We retrospectively reviewed cytological preparations of 678 nodules that were originally identified as CFO nodules, including conventional specimens in 209 nodules, liquid based cytology (LBC) specimens in 221 nodules, and both conventional and LBC specimens in 248 nodules. Ultrasound reports with representative photographs were also reviewed.

**Results:**

Of the 678 CFO nodules, 214 (31.6%) were reclassified into other categories, including non‐diagnostic/unsatisfactory (ND/UNS) except for CFO (n = 15), benign (n = 198), and malignant (n = 1). Conventional preparations (33.5%) were more frequently reclassified than LBC preparations (13.6%; *P* < .0001). Re‐aspiration for diagnosis was performed for only one calcified nodule. The rates of surgical resection and malignancy were 3.0% and 0.2%, respectively. Based on American Thyroid Association guidelines and the Kuma Hospital ultrasound classification, worrisome sonographic features were identified in 5.8% and 0% of CFO nodules, respectively.

**Conclusion:**

We propose that CFO nodules should be classified as separate from ND/UNS nodules; they should be categorized as a subtype of benign nodules. However, it is essential that fine‐needle aspiration cytology be performed under ultrasound‐guided real‐time visualization of needle placement in the target nodule in all cases.

## 1 INTRODUCTION

According to The 2017 Bethesda System for Reporting Thyroid Cytopathology (TBSRTC),^1^ the diagnostic categories for thyroid nodules are classified as “non‐diagnostic or unsatisfactory (ND/UNS),” “benign,” “atypia of undetermined significance (AUS),” “follicular neoplasm (FN),” “suspicious for malignancy (SFM),” and “malignant,” and the risk of malignancy (ROM) and recommended clinical management are specific to each category. ND/UNS applies to specimens that are unsatisfactory owing to the following: (a) fewer than six groups of well‐preserved, well‐stained follicular cell groups with 10 cells each; (b) poorly prepared, poorly stained, or obscured follicular cells; or (c) cyst fluid, with or without histiocytes, and fewer than six groups of 10 benign follicular cells. As the possibility of cystic papillary thyroid carcinoma cannot be excluded in the last scenario, namely cyst fluid only (CFO) nodules, the cases are classified as ND/UNS.^1^, ^2^ However, in Japanese Society of Thyroid Surgery (RSJSTS) 2015 reporting system, CFO nodules were not categorized under ND/UNS but as an independent diagnostic category.^3^

The clinical value of CFO nodules depends largely on sonographic correlation.^1^, ^4^ When the nodule is a simple cyst without worrisome sonographic features, an endocrinologist might proceed as if it is a benign nodule. Alternatively, when the sonographic features suggestive of malignancy, the endocrinologist is not convinced that the sample is representative, and it might be clinically ND/UNS.^5^ Thus, CFO nodules have two different clinical managements that can be determined based on ultrasound findings. To date, the availability of extensive research focusing on CFO nodules is limited,^6^, ^7^, ^8^ and the ROM in CFO nodules has not been well established. The purpose of the current study was to review cytological and ultrasound findings of CFO nodules, define the ROM, and determine the recommended clinical management of CFO nodules.

## 2 MATERIALS AND METHODS

We reviewed a database of the cytology reports of 7295 patients that underwent thyroid fine‐needle aspiration cytology (FNAC) at Kuma Hospital from January to December 2017. During this period, cytology specimens from 9767 thyroid nodules were available. Of these, 678 (6.9%) were reported as CFO nodules, based on the criteria of TBSRTC.^1^ Most of the aspirations were performed using a 22‐gauge needle, and an 18‐gauge needle was used to drain cyst fluid. The procedure was done under ultrasound‐guided real‐time visualization of needle placement in the target nodule in all cases. The aspirated materials were prepared using conventional methods, liquid‐based cytology (LBC), or both. The conventional smears were produced using a press and release method,^9^ and were fixed with Cytorop (Alfresa, Japan), which is a cytological fixative. When excessive fluid was aspirated, the fluid components were removed by tilting the preparations, smears were done using the press and release method, and preparations were fixed 10 to 20 seconds after smearing.^9^ For LBC, CytoRich RED collection fluid (Becton, Dickinson and Company, New Jersey) with haemolytic and proteolytic abilities was used for the CytoRich hand method.^10^ Both preparations were stained with Papanicolaou stain. Thereafter, the conventional specimens in 209 nodules, LBC specimens in 221 nodules, and both conventional and LBC specimens in 248 nodules were retrospectively reviewed. We also reviewed previous ultrasound findings saved in electronic medical records. Ultrasound was performed using the APLIO 80 SSA‐770A (Toshiba Medical Systems Co., Ltd., Otawara, Japan) or the APLIO 500 TUS‐A500 (Toshiba) with the PLT‐805AT (Toshiba) or PLT‐1005BT (Toshiba) probe. The ultrasound reports were interpreted based on both the Kuma Hospital ultrasound classification (KH‐USC), previously described by Ito et al,^11^, ^12^ and the American Thyroid Association (ATA) guidelines.^4^ The KH‐USC classifications 5 and 4, 3, 2, and 1 roughly correspond to the high suspicion, intermediate suspicion, low suspicion, very low suspicion, and benign categories outlined in the ATA guidelines, respectively. The study was conducted in accordance with the guidelines of the ethics committee of Kuma Hospital; approval was granted by the ethic committee, and all subjects provided informed consent. The statistical significance of the data was determined using the Fisher's probability test, and *P* < .05 was considered statistically significant.

## 3 RESULTS

Table 1 shows the revised classification of the 678 nodules originally reported as CFO nodules. In the re‐evaluation, 464 patients remained in the same category, and the remaining 214 (31.6%) nodules were reclassified into other categories, including 15 (2.2%) in ND/UNS excluding CFO (ND‐other), 198 (29.2%) in benign, and 1 (0.1%) in malignant. The reasons for being reclassified into ND‐other and benign were obscure fluid materials and the presence of adequate follicular cells, respectively. In one malignant nodule, a few papillary thyroid carcinoma cells were present in both conventional and LBC preparations. No nodules were reclassified into AUS, FN, or SFM. Conventional preparations (70 nodules, 33.5%) were more frequently reclassified into other categories than LBC preparations (30 nodules, 13.6%) (*P* < .0001).

**Table 1:** Revised classification of 678 nodules originally reported as cyst fluid only

	Conventional	LBC	Conventional and LBC	Total
ND/UNS	152 (72.7%)	192 (86.9%)	135 (54.4%)	479 (70.6%)
ND‐other	13 (6.2%)	1 (0.5%)	1 (0.4%)	15 (2.2%)
	*P* = 0.0016			
CFO	139 (66.5%)	191 (86.4%)	134 (54.0%)	464 (68.4%)
	*P* = 0.0784			
Benign	57 (27.3%)	29 (13.1%)	112 (45.2%)	198 (29.2%)
	*P* = 0.0031			
AUS	0 (0%)	0 (0%)	0 (0%)	0 (0%)
FN	0 (0%)	0 (0%)	0 (0%)	0 (0%)
SFM	0 (0%)	0 (0%)	0 (0%)	0 (0%)
Malignant	0 (0%)	0 (0%)	1 (0.4%)	1 (0.1%)
Total	209	221	248	678

Abbreviations: AUS, atypia of undetermined significance; CFO, cyst fluid only; FN, follicular neoplasm; LBC, liquid based cytology; ND‐other, nondiagnostic or unsatisfactory excluding cyst fluid only; ND/UNS, nondiagnostic or unsatisfactory; SFM, suspicious for malignancy.

Table 2 shows original ultrasound classifications and clinical outcomes of CFO nodules. Among 469 CFO nodules, 18 (3.8%) were re‐aspirated. The incidences of the re‐aspiration in KH‐USC categories 4, 3, 2, and 1 were 100%, 0%, 4.0%, and 0%, respectively. Re‐aspiration was performed to facilitate diagnosis for only one calcified nodule in which malignancy was suspected by ultrasound. The second aspiration was performed 7 days after initial aspiration. In 11 KH‐USC 3 nodules, re‐aspiration was not done. In the remaining 17 nodules, cytological examination was combined with drainages of fluid or percutaneous ethanol injection therapy (PEIT). The duration between the initial and second aspirations was less than 2 weeks in 2 nodules, 2 weeks to 3 months in 5 nodules, and more than 3 months in 11 nodules. Cytological diagnoses of re‐aspiration included CFO (13 nodules, 72.2%), benign (4 nodules, 22.2%), and AUS (1 nodule, 5.6%).

**Table 2:** Original ultrasound classification and clinical outcome of 469 cyst fluid only nodules

KH‐USC	Nodules 469 (100%)	Re‐aspiration 18 (3.8%)	Indication	Duration (*n*)	Cytology (*n*)	Resection 14 (3.0%)	Indication	Histologic diagnosis (*n*)
4.0	1 (0.2%)	1 (100%)	US: PC, suspected	<2 weeks	AUS (1)	0 (0%)	None	None
3.0	11 (2.3%)	0 (0%)	None	None	None	1 (7.1%)	Combined with other coexisting nodule (1)	Benign nodular goitre (1)
2.0	422 (90.1%)	17 (4.0%)	Drainage (7)	>3 months (6)	CFO (4) Benign (2)	11 (78.6%)	Combined with other coexisting nodule (7)	Benign nodular goitre (6)
				< 2 weeks (1)	Benign (1)			WDT‐UMP (1)
			PEIT (10)	> 3 months (5)	CFO (4) Benign (1)		Large nodule (4)	Benign nodular goitre (3)
				> 2 weeks, < 3 months (5)	CFO (5)			FT‐UMP (1)
1.0	32 (6.8%)	0 (0%)	None	None	None	1 (7.1%)	Combined with other coexisting nodule (1)	Benign nodular goitre (1)
Unclassified	3 (0.6%)	0 (0%)	None	None	None	1 (7.1%)	US: Malignancy could not be excluded	FC (1)

*Note*: *n* indicates number of nodules.

Abbreviations: AUS, atypia of undetermined significance; CFO, cyst fluid only; FC, follicular carcinoma; FT‐UMP, follicular tumor uncertain malignant potential; KH‐USC, Kuma Hospital ultrasound classification; PC, papillary carcinoma; PEIT, percutaneous ethanol injection therapy; US, ultrasound; WDT‐UMP, well‐differentiated tumor uncertain malignant potential.

Fourteen CFO nodules (3.0%) were surgically resected during the follow‐up period from 1.5 to 2.5 years and underwent histological examination. Among them, nine nodules were resected concurrently with coexisting nodules, four were resected because of their large size, and the remaining one nodule underwent surgery because malignancy could not be excluded by ultrasound. The histological diagnoses included benign nodular goiter (11 nodules), follicular tumor of uncertain malignant potential (1 nodule), well‐differentiated tumor of uncertain malignant potential (1 nodule), and follicular carcinoma (1 nodule). The incidence of malignancy was 0.2% (1/469) for CFO nodules and 7.1% (1/14) for resected CFO nodules.

Table 3 showed the revised ultrasound classifications of CFO nodules based on the ATA guidelines and KH‐USC. In total, 84.6% (397 nodules) and 98.5% (462 nodules) of CFO nodules were interpreted as benign lesions according to ATA guidelines and KH‐USC, respectively. Based on ATA guidelines, 27 CFO nodules (5.8%) exhibited high or intermediate suspicion, for which re‐aspiration was recommended, due to the presence of microcalcifications, rim calcifications with a small extrusive soft tissue component, or hypoechoic solid nodules with smooth margins and without microcalcifications. In contrast, we identified no nodules identified as KH‐USC 5 or 4 that were consistent with the suspected categories.

**Table 3:** Revised ultrasound classification of 469 cyst fluid only nodules

ATA	High suspicion	Intermediate suspicion	Low suspicion	Very low suspicion	Benign	Total
KH‐USC						
5	0	0	0	0	0	0
4	0	0	0	0	0	0
3	4	1	0	0	0	5
2	15	5	45	381	0	446
1	0	0	0	0	16	16
Unclassified	2	0	0	0	0	2
Total	21	6	45	381	16	469

Abbreviations: ATA, American Thyroid Association; KH‐USC, Kuma Hospital ultrasound classification.

## 4 DISCUSSION

According to the ATA guidelines and TBSRTC,^1^, ^4^ preparations exhibiting degenerated cyst fluid contents, with or without histiocytes, and fewer than six groups of 10 benign follicular cells are classified as ND/UNS, because the possibility of cystic papillary thyroid carcinoma cannot be excluded. Abundant hemosiderin‐laden macrophages do not count toward specimen adequacy. Similarly, the UK Royal College of Pathologists^13^ and the Italian Society of Anatomic Pathology and Diagnostic Cytology^14^ handle CFO nodules as ND/UNS nodules, that is, Thy1c (non‐diagnostic for cytological diagnosis‐cystic lesion) and TIR1C (non‐diagnostic/cystic), respectively. Conversely, in Japan, the CFO nodule is not included in the ND/UNS category but is handled as an independent diagnostic category.^3^

In this study, we showed that 31.6% of nodules originally classified as CFO were reclassified into other categories. Most of them had adequate numbers of benign follicular cells and were consistent with a benign classification. Jaragh et al reported similar findings.^7^ They found that 22 of 76 CFO nodules should have been identified as having adequate benign follicular cells during retrospective review. The findings indicated that there is large observer variability in the diagnosis of CFO nodules, and the proportion of CFO nodules identified from thyroid FNAC might be relatively low. This has implications for the ROM of CFO nodules. In addition, conventional preparations were more frequently reclassified into ND‐other and benign than into LBC preparations (*P* < .0001). On conventional preparations from fluid materials, follicular cells tended to be overlooked more often than they are with LBC preparations. It is well‐recognized that LBC is useful for reducing the number of unsatisfactory specimens,^10^, ^15^ and our findings confirmed the view. In addition, the preparation methods seem to influence the incidence and the ROM of CFO nodules.

As CFO nodules are mostly benign and rarely undergo surgical resection, it is difficult to estimate the ROM accurately. The ROM of CFO nodules have reportedly ranged from 0% to 14.3%.^6^, ^16^, ^17^, ^18^ Güney and Şahiner reported that, in their study, malignancy was not detected in any of the specimens taken from 14 out of 48 patients who were diagnosed with CFO nodules and had undergone operations.^8^ According to a report by Jaragh et al, of the 76 CFO cases with subsequent thyroidectomy, 10 cases (13.2%) had an ipsilateral diagnosis of papillary carcinoma measuring ≥1.0 cm.^7^ Takada et al. reported that the ROM of nodules classified as CFO and ND‐other were 2.0% and 5.6%, respectively, and differed significantly (*P* < .01). In the current study which was performed in the same hospital, 3.0% of CFO nodules were surgically resected, and the ROM was 0.2% for CFO nodules and 7.1% for resected CFO nodules. The ROM (0.2%) of the present study was strikingly lower than that (2.0%) of 10 years ago. The difference may be due to the development of ultrasound, improvement of aspiration skills and diagnostic ability, combined use of the LBC method, and a short follow‐up period.

In countries otherthan Japan, CFO nodules seem to beclassified as ND/UNS because thepossibility of cystic papillary thyroid carcinoma cannot be excluded. When a nodule is entirely cystic and has no worrisome sonographic features, it is handled as a benign nodule. On the other hand, when the sonographic features are worrisome, it is considered clinically unsatisfactory. It is important to ascertain whether these classifications and handling strategies are appropriate.

If we think that cases diagnosed as CFO or benign should not contain malignancy, we will not be able to use the terms “CFO” or “benign” as diagnostic categories in the cytology. Thus, this concept is not practical. The presence of a few malignant cases should be allowed in cases diagnosed as CFO or benign. The problem is then defining the permissible ROM. This decision may involve consideration of both academic data and the social environment. In our study, the ROM of CFO nodules was 0.2%. The incidence was significantly lower than that of ND‐other nodules. The ROMs of nodules diagnosed as benign was 1.1%, and 13.7% for subsequently resected benign nodules.^19^ The ROMs of CFO nodules are evidently lower than those of benign nodules. In Japan, the fact that CFO nodules reveal such lower ROMs indicates that these cases should not be handled as ND/UNS.^3^ Based on ATA guidelines, worrisome sonographic features were identified in 5.8% of CFO nodules. Based on KH‐USC, no CFO nodules with worrisome sonographic features were detected. As per the RSJSTS reporting system, we suggest that there is no reason to categorize CFO nodules with an extremely low ROM as ND/UNS. In addition, CFO and ND‐other nodules differ in terms of their clinical management.^1^, ^4^ For benign nodules, the ATA guidelines recommend that follow‐up should be determined by risk stratification based on ultrasound patterns.^4^ We propose that CFO nodules should be classified separately from ND/UNS nodules; They should be classified as an independent category, similar to that in Japan,^3^ or a subtype of benign nodules. However, it is essential that FNAC be performed under ultrasound‐guided real‐time visualization of needle placement in the target nodule in all cases. Our proposal is based on the data collected from a thyroid center with high patient volume in Japan. Therefore, further research analyzing data from other hospitals and other countries is warranted.

## CONFLICT OF INTEREST

The authors declare no conflicts of interest.

## FINANCIAL DISCLOSURE

The authors have no connection to any companies or products mentioned in this article.
